# Anthropometric analysis of the relationship between mandibular biotypes and condylar fracture patterns and degree of condylar displacement in victims of facial trauma

**DOI:** 10.1007/s10006-026-01576-y

**Published:** 2026-05-23

**Authors:** Eduardo Luis de Souza Cruz, Christian Ruben Cusi Fernandez, José Thiers Carneiro Junior

**Affiliations:** Federal University of Pará. Rua Augusto Correa, 01. CEP 66075-110. Guamá, Belém, Pará, Brazil

**Keywords:** Anthropometry, Mandibular condyle, Tomography, Condyle fracture

## Abstract

**Objectives:**

This project aimed to investigate the relationship between gender, age, mandibular biotype, condylar fracture patterns, and their degree of displacement.

**Material and methods:**

To this end, a sample of 200 facial trauma patients was analyzed to collect data such as age, gender, condylar fracture type, and the degree of displacement. Furthermore, computed tomography scans were analyzed to define mandibular biotypes and the degree of displacement of the condylar bone stumps.

**Results:**

There was a strong and statistically significant association between mandibular biotype and the type of condylar fracture (p < 0.001). It is likely that the gender variable is associated with mandibular biotype in the analyzed sample, with V-shaped mandibles more common in women (p = 0.0004), and smaller fracture displacement angles (p = 0.01) due to a greater tendency for high fractures (p = 0.01).

**Conclusions:**

There is relationship between mandibular biotype and its role as a marker of sexual dimorphism, in addition to its contribution to the degree of displacement and type of condylar fractures in patients who have suffered facial trauma.

**Clinical relevance:**

Mandibular structure, such as shape, height, and width; bone density, cortical and medullary volume; and the mechanical action of the masticatory muscles can affect the variability, stability, and location of fractures, interfering with the response to the magnitude, dissipation, and direction of external mechanical forces originating from the energy of the trauma. Anthropometric study of anatomical features can provide relevant data for better forensic understanding. Regarding the mandible, it can be considered an important tool for understanding mandibular trauma, influencing diagnosis and treatment decision-making.

## Introduction

The mandible is the largest and most resistant facial bone, and also the most frequently affected by trauma. Of facial fractures, mandibular fractures are the most common, accounting for rates ranging from 12 to 56% of cases; of these, approximately 29% to 52% of these mandibular fractures are condylar [1, 2].

The main etiological factors of mandibular condyle fractures consist of falls, physical assaults, and car accidents, showing a higher rate of occurrence among young men, aged between 20 and 40 years. Age, gender, and severity of trauma, with or without soft tissue damage, are strongly linked to the incidence of this type of fracture [2–5].

The mandibular structure, such as shape, height, width; bone density, cortical and medullary volume, and the mechanical action of the masticatory muscles are intrinsic elements that can affect the variability, stability, and location of fractures, interfering with the response to the magnitude, dissipation, and direction of external mechanical forces arising from the energy of the trauma. Despite this, many studies do not point to these anatomical aspects as predisposing factors to mandibular trauma [1, 2, 6, 7].

Mandibular anthropometry represents a fundamental field of study in forensic dentistry and anthropology, providing valuable information on the morphological characteristics of the mandible and its variations related to age, gender, and facial trauma patterns. Several studies have used specific anthropometric parameters for mandibular analysis through panoramic radiographs (Orthopantomography), considering parameters such as Coronoid Ramus Height (CRM); Condylar Ramus Height (CRM); Projective Ramus Height (PRH); Minimum Ramus Width (MRW); Gonial Angle (GA); Bigonial Width (BGW); Antegonial depth (Ant.D) and Antegonial angle (Ant.A) [8–13].

The relationship between mandibular anthropometry and facial fracture patterns is a crucial aspect for understanding trauma mechanisms and developing preventive and therapeutic strategies, highlighting forensic applications, planning surgical approaches, risk assessment, and the possibility of developing predictive models; especially in underdeveloped centers lacking access to adequate imaging exams. Despite this, population variability, the influence of genetic, environmental, and nutritional factors; The low availability of specific population data in epidemiological databases, in addition to studies based on panoramic radiographs, are real limitations for a better anthropometric understanding of the mandible in facial trauma [8–13].

In this way, this study investigated the correlation between mandibular biotype, the degree of displacement of fractured mandibular condyles, and the respective fracture patterns in a sample of patients from northern Brazil using three-dimensional tomographic analysis. The identification of these correlations, even in a primary way, can provide fundamental data for the development of more effective treatment strategies, especially in complex or multiple fractures, such as those involving the mandibular condyle and other regions of the mandible.

## Materials and methods

### Type of study

An observational, descriptive, and cross-sectional study with a quantitative approach was conducted. The research was developed from the collection of data from 200 medical records of patients victims of car/motorcycle accidents and diagnosed with condylar fractures treated at a reference Emergency Hospital in Belém, Pará, Brazil.

### Ethical aspects

This Research Project was evaluated by the Research Ethics Committee of the Ophir Loyola and State University of Pará,, Belém, Pará, Brazil, to obtain favorable opinion no. 7,761,086 (Annex), which enabled the analysis and publication of the results. Medical records of patients treated in the last 4 years (2021–2024) by the Oral and Maxillofacial Surgery and Traumatology Service of the Metropolitan Emergency Hospital in Belém, Pará, Brazil were analyzed. This study was conducted in accordance with current ethical principles and the 1964 Helsinki Declaration.

### Inclusion and exclusion criteria

Included were medical records of patients over 18 years of age; both sexes; diagnosed with unilateral condylar fractures confirmed by clinical and tomographic examination available in electronic medical records; and who have a signed Informed Consent Form (ICF).

Excluded from this study were minors, incomplete electronic medical records; patients with a history of previous mandibular surgery that may alter condylar measurements, such as mandibular reconstructions, Orthognathic Surgery and Temporomandibular Joint (TMJ) Surgery; patients with others mandibular fractures; patients with congenital deformities or syndromes; and those who have not signed the Informed Consent Form (ICF).

### Data collection

#### Sample profile analysis

The following qualitative variables were considered for sample characterization: Gender (1: Male; 2: Female); Age (numeral); and Type of mandibular condyle fractures (1: Low—Extracapsular; 2: High—Intracapsular), according to the criteria of Eckelt et al. [14] and the current AO/CMF Comprehensive Classification System.

#### Quantification of the degree of displacement of condylar fractures

**Fig. 1:**
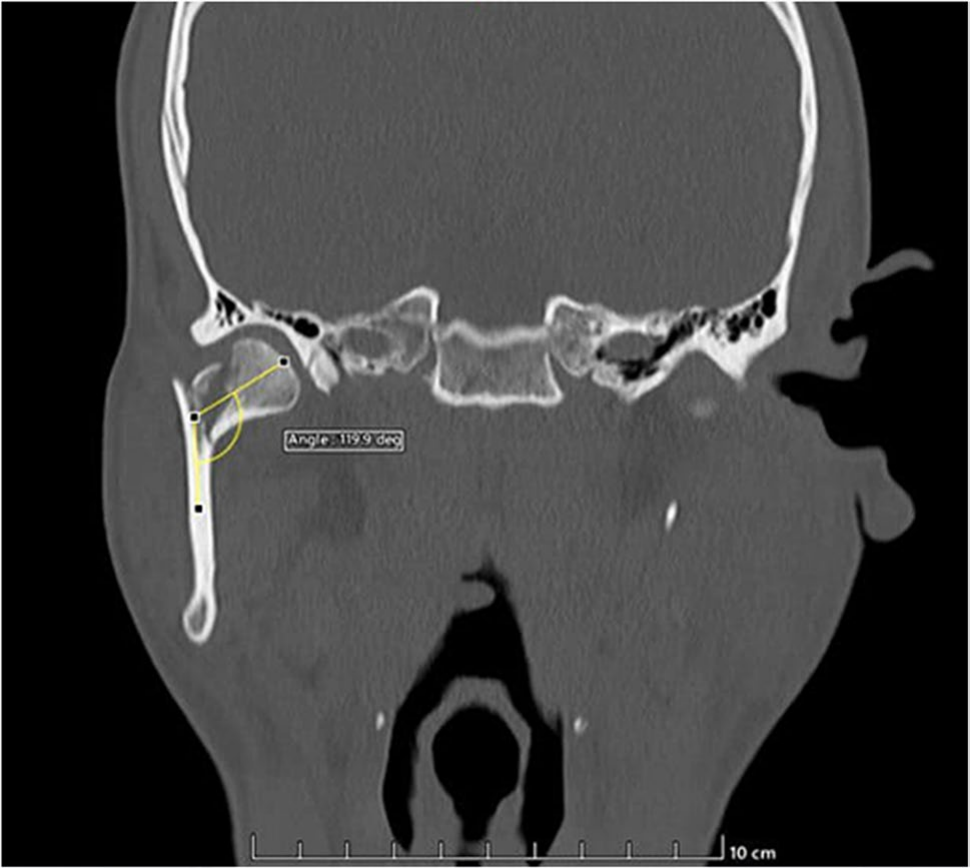
Methodology: Quantification of the Degree of Displacement of Condylar Fracture Bone Stumps. In Facial Computed Tomography, coronal slices containing the most central portions of the posterior margin of the mandibular ramus and the fractured condylar fragment are selected. Thus, two straight lines are projected (yellow color) as virtual extensions of the fractured bone stumps for quantification of the obtuse angle between them using the “Angle tool”, ImageJ software version 18 (National Institutes of Health, Bethesda, Maryland, USA)

Tomographic images were analyzed in coronal views, with the center of the fractured condyles and the posterior portion of the mandibular ramus as anatomical reference points for standardization of tomographic slices. Using ImageJ v. software. 18 (National Institutes of Health, Bethesda, Maryland, USA), two straight lines were projected (yellow color) as virtual extensions of the fractured bone stumps, with the angle between them quantified using the “Angle tool” (Fig. 1). These measurements were performed for each patient in triplicate by 03 different operators, adopting the average as the individual result.

#### Determination of mandibular biotypes

**Fig. 2:**
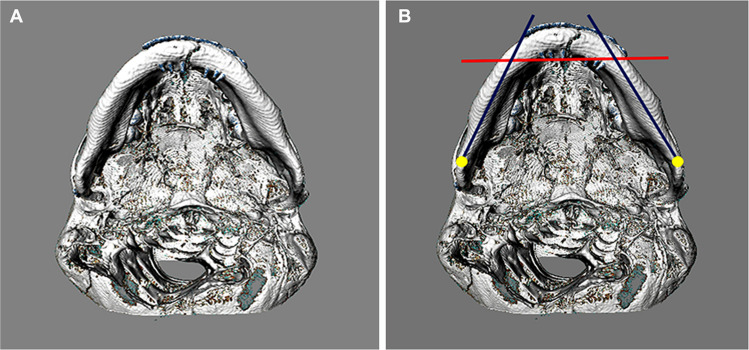
Methodology: Determination of the Mandibular Biotype. (**A**) Three-dimensional reconstructions of the mandibles were analyzed in caudal view, aligning the midline and identifying the bilateral mentoforms. (**B**) Three lines are projected, two starting from the cephalometric point Gonion (Go) on each side, along the mandibular base, passing through the ramus and body in blue; One line (red) is projected crossing the previous ones, tangent to the posterior walls of the bilateral mandibular canals. Using the “Angle tool” (Software ImageJ v. 18, National Institutes of Health, Bethesda, Maryland, USA) the two angles formed by them are quantified and the arithmetic mean is calculated

Three-dimensional reconstructions of the mandibles were analyzed using ImageJ v. 18 software (National Institutes of Health, Bethesda, Maryland, USA) in caudal views. Two straight lines (blue color) were projected coinciding with the center of the mandibular base along the entire mandibular perimeter starting from the Gonion point (Go), bilaterally. Additionally, a line (red color) was projected connecting the posterior margins of the mental foramina, so as to cross the basal lines (blue). The angle formed by them was quantified using the “Angle tool” (Fig. 2). These measurements were performed on both sides for each patient and in triplicate by 03 different operators, adopting the average as the individual result.

The biotypes were categorized in two ways: obtuse angles less than or equal to 135º were considered closed, resulting in U-shaped jaws (Score 1); obtuse angles greater than 135º were considered open, resulting in V-shaped jaws (Score 2).

### Statistical analysis

The data were tabulated and analyzed using Bioestat 5.0 statistical software (Institute Mamirauá). Descriptive analyses (medians, absolute and relative frequencies) were performed to characterize the sample. To verify the correlation between the parametric variables, Spearman's Correlation tests were applied due to the abnormality of the data; as well as Simple Linear Regression. For non-parametric and dichotomous variables, Pearson's correlation test and Chi-square test were used to investigate the association between these variables.

## Results

### Sample profile regarding gender, age, mandibular biotype, fracture types, and degree of displacement

This study involved collecting information from 200 patients who were victims of facial trauma and diagnosed with unilateral condylar fractures. Of these, 167 patients were male, representing 83.5% of the sample, with a median age of 32 years. Approximately 95.5% of the patients were identified with U-shaped mandibular biotypes, with median angles of 117.4 degrees. 69.5% of the patients presented with high-type fractures (intracapsular). 30.5% of the patients were affected by low fractures with a degree of displacement around 146.1° (Table 1).

**Table 1:** Descriptive analysis of the variables Gender, Age, Mandibular Biotype, Type of Condylar Fracture and Degree of Fracture Displacement

	Absolute frequency	Relative frequency	Median
Gender			
Male	167	83,5%	
Female	33	16,5%	
Mandibular biotype			
U-shaped	191	95,5%	
V-shaped	9	4,5%	
TYPE OF CONDYLAR FRACTURE			
Low	61	30,5%	
High	139	69,5%	
Age (Years)	-	-	32
Mandibular Biotype (Degrees)	-	-	117,4°
Degree of fracture Displacement	-	-	146°

**Fig. 3:**
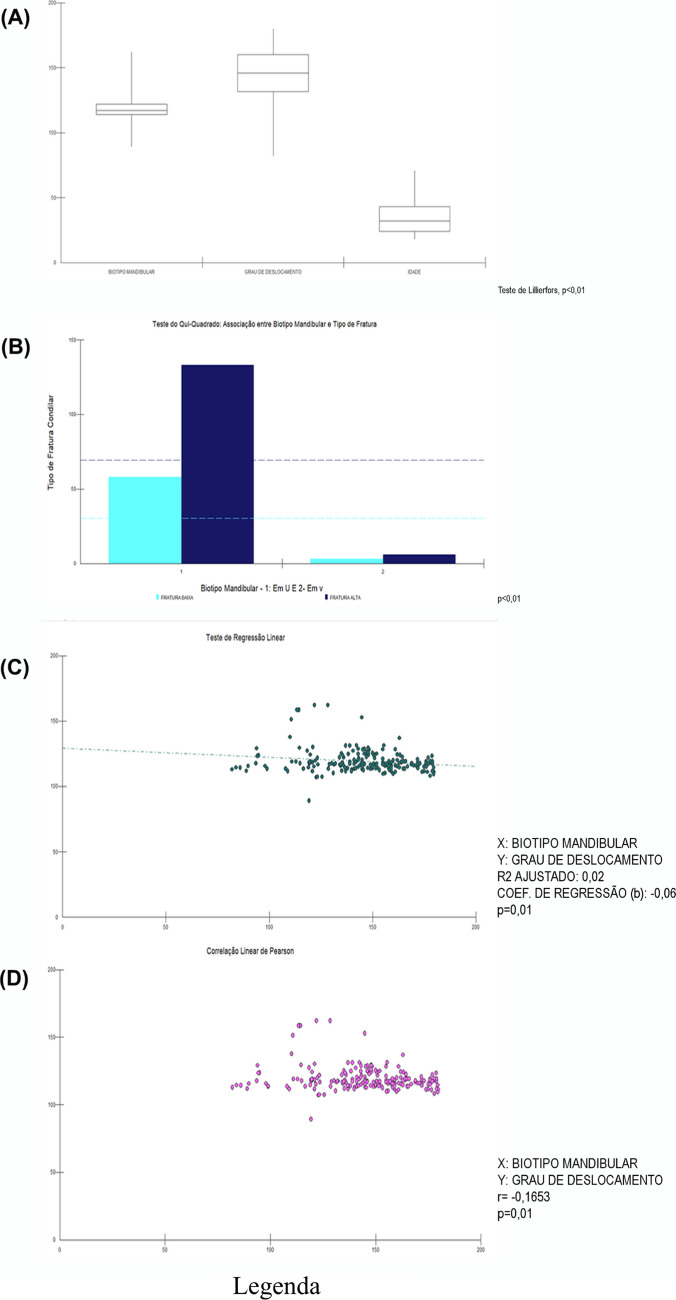
Methodology: Statistical Analysis Graphs. (**A**) Boxspot: Distribution of data referring to quantitative variables mandibular biotype, Degree of Displacement and Age. The descriptive analysis of the parametric data revealed abnormal data behavior due to sample variability, resulting in asymmetry in the distribution (LILLIEFORS TEST, p < 0.01). (**B**) Columns: When analyzing the results of the Scores used to categorize mandibular types as U or V in relation to High and Low types of condylar fractures, it is possible to identify agreement in the frequencies between the variables studied (Chi-square, p < 0.001). (**C**) and (**D**) Scatter Plots: association between Mandibular Biotype and Degree of Displacement. The majority of the sample, represented in pairs by the points, shows concentration along the slightly inclined trend line (b < 0), demonstrating an inverse proportion between the variables. The greater the angle related to the biotype, the lower the degree of displacement of the fractured stumps (p < 0.05). It is possible to observe the presence of outliers, which clearly shows the sample heterogeneity

The parametric data showed an abnormal distribution, with p < 0.01. Thus, medians were adopted for Age, Mandibular Biotype—Degrees (median of 117.4°) and Degree of Fracture Displacement with a median of 146.1°. The data distribution pattern can be observed in graph A, Fig. 3.

### Association between mandibular biotype and condylar fracture type

When analyzing the results of the Scores used to categorize mandibular types as U or V in relation to High and Low types of condylar fractures, it is possible to identify agreement in the frequencies between the variables studied (Chi-square, p < 0.001). This result allows us to conclude that there is a strong and statistically significant association between mandibular biotype and condylar fracture type (Table 2 and Graph B, Fig. 3).

**Table 2:** Association between mandibular biotype and condylar fracture type

Type of condylar fracture	Low	Hight	Total
U-shaped	58	133	191
V-shaped	3	6	9
Total	61	139	200

Note: Chi-square test, with p < 0.01 for absolute frequency values ​​considering Scores 1 and 2 for U and V Mandibular fractures, respectively. And Scores 1 and 2 for Low and High Fracture types, respectively

### Relationship between age and the incidence of mandibular biotype, degree of displacement and fracture types

During the data analysis, it was not possible to identify a statistically significant difference regarding the role of the age variable as a relevant factor for determining the mandibular biotype (p = 0.40), the degree of displacement (p = 0.60), and the type of fracture (p = 0.95) (Table 3).

**Table 3:** Relationship between age, mandibular biotype, type of condylar fractures and the degree of displacement in patients with condylar fractures

Parameters	Regression Coeff. (b)	P
Mandibular Biotype	0,04	0.40
Degree of Fracture Displacement	−0,06	0,60
Type of Condylar Fractures (Low/Hight)	0,02	0,95

Note: Simple Linear Regression, adopting p less than or equal to 0.05. Dependent variable: Age. (*) Statistical significance

### Relationship of gender with the incidence of mandibular biotype, degree of displacement, and types of condylar fracture

Our results demonstrate that the gender variable is correlated with the determination of the mandibular biotype (p = 0.0004), with a positive Spearman coefficient (rs) equal to 0.2465, considered weak. This result allows us to affirm that there is an increasing correlation between the two variables, rejecting the null hypothesis, being statistically significant and not a product of mere chance. Thus, it is likely that the gender variable is associated with the mandibular biotype in the analyzed sample, with V-shaped mandibles more present in women. On the other hand, gender did not show statistical significance in relation to the type of condylar fracture (p = 0.42) with a negative Spearman coefficient (rs) close to zero. As for the Degree of Displacement, rs = −0.10 was observed as a probable slight negative association, but without a statistically significant difference (p = 0.13) (Table 4).

**Table 4:** Association between Gender, mandibular biotype, degree of fracture displacement, and types of condylar fractures in a patient who was a victim of facial trauma

Parameters	Spearman Coeff. (rs)	P
Mandibular Biotype	+ 0,24	0.0004*
Degree of Fracture Displacement	−0,10	0,13
Type of Condylar Fractures (Low/Hight)	−0,05	0,42

Note: Spearman correlation, adopting p less than or equal to 0.05. Dependent variable: Gender

(*) Statistical significance

### Role of mandibular biotype in the degree of displacement of fractured fragments

Through Linear Regression it was possible to understand the potential determinant of the mandibular biotype in the degree of displacement of condylar fractures, showing statistical significance of p = 0.01 with a Regression Coefficient (b) of −0.06. These findings demonstrate a probable and slight inversely proportional trend between mandibular angles and fracture displacement degrees, in a ratio that the larger the angle of the mandibular biotype, and the greater the tendency for V-shaped mandibles, the smaller the angles of condylar fracture displacement would be (Fig. 3, Graph C). The presence of outliers in the sample, interfering with sample homogeneity, makes more concrete conclusions difficult despite the level of significance. It is also possible to estimate the value of the condylar displacement angle through Pearson's Linear Correlation, p = 0.01 (Fig. 3, C and D).

### Potential determinant of the mandibular biotype for the type of condylar fracture

The low or high incidence of condylar fractures can be determined by the mandibular biotype in a positive and significant ratio (Regression Coefficient = + 8.86, with p = 0.01). Thus, based on the sample analyzed, it is possible to state that the larger the angle of the mandibular biotype, the greater the tendency for high fractures to occur (Table 5).

**Table 5:** Trend model for determining the type of condylar fracture in relation to the mandibular biotype

Parameters	Regression Coeff. (b)	P
Type of Condylar Fractures (Low/Hight)	+ 8,86	0,01*

Note: Simple Linear Regression, adopting p less than or equal to 0.05. R^2^ = 0.02

Dependent variable: Mandibular Biotype

(*) Statistical significance

## Discussion

The mandible is an intramembranous bone, belonging to the lower third of the face, which presents strong particularities such as size, shape, resistance, and force dissipation mechanics. In addition, it is commonly involved in facial trauma with rates ranging from 12 to 56% of cases; of these, approximately 29% to 52% of these mandibular fractures are condylar [1–5]. Other studies indicate an incidence rate of condylar processes ranging from 30 to 50% of cases, with 80% affecting only one side of the condyles [15].

Condylar process fractures mainly affect males in the young age range of 20 to 40 years. The main etiological factors are falls, physical assaults, and car accidents. 72% of car accidents affect the head region and can result in fractures, which generates higher health costs, time off work, and lost productivity [2–5, 15].

Our sample is mostly composed of men—83.5%—young—around 32 years old—victims of traffic accidents, with unilateral condylar fractures associated or not with other facial fractures, corroborating the data present in the literature. Since this is a preliminary anthropometric study and given the need to simplify data analysis in studies of this type, the authors chose to include only unilateral fractures in the sample, consistent with the higher incidence of cases described in the literature.

Other factors such as gender and severity of trauma, with or without soft tissue damage, are strongly linked to the incidence of this type of fracture and are well elucidated in the scientific literature. [2–5]. In addition, characteristics such as shape, height, width,bone density, cortical and medullary volume, the mechanical action of the masticatory muscles and the presence or absence of teeth are intrinsic elements that can affect the variability, stability and location of fractures, interfering with the response to the magnitude, dissipation and direction of external mechanical forces arising from the energy of the trauma. Despite this, many studies do not point to these anatomical aspects as predisposing factors to mandibular trauma, nor do they seek diagnostic determination models with three-dimensional examinations [1–5].

Patients who are victims of facial trauma and have condylar fractures present with pain, dysphagia, dysphonia, limited mouth opening, altered mandibular movements with bone crepitus, and dental malocclusion. In this sense, anthropometric knowledge can significantly influence clinical and forensic practices, as demonstrated in studies that address the relevance of complete classification systems for mandibular fractures based on anatomical particularities. Diagnosing and making accurate treatment decisions is crucial for functional recovery, bone and dental occlusion restoration, as well as recovery of the Temporomandibular Joints [10, 15–29].

Mandibular anthropometry demonstrates consistent findings in the literature regarding significant sexual dimorphism in relation to mandibular shape. These characteristics show that in male individuals the mandible has larger dimensions in shape and robustness, wider mandibular rami, more prominent and acute angles, and greater chin prominence. Furthermore, the gonial angle (GA), coronoid ramus height (CNH), and projective ramus height (PRH) show positive coefficient functions for sex determination [30–33]. Our results corroborate the findings of the main anthropometric data described, making it possible to affirm that the mandibular biotype can likely be considered a predictor of sexual dimorphism (p = 0.0004), as are other characteristics already elucidated in the literature. Thus, women would tend to have more open biotypes in this sample of facial trauma. However, our data on sexual dimorphism contrast with other studies that do not point to sexual differences considering the shape of the mandibular base, not the angle analyzed in this study [31–35].

This preliminarily study performed an anthropometric analysis of the mandible in patients with condylar fractures and, using tomographic examinations, sought to establish a correlation model. The authors make it clear that the population studied consists of Brazilian nationals of mixed race, belonging to the Amazon region, characterized by strong indigenous influences. Therefore, this is a specific group that cannot be subject to absolute international generalizations, but it corroborates the results of studies in other populations around the world.

## Final considerations

It is possible to verify a relationship between mandibular biotype as a marker of sexual dimorphism, in addition to contributing to the degree of displacement and type of condylar fractures in patients who are victims of facial trauma. Thus, it is likely that the mandibular shape contributes to the dissipation of forces in facial trauma, interfering with the diagnosis. In this sense, the analysis of the mandibular biotype can be considered a characteristic of clinical relevance.

This study is a preliminary analysis of the intrinsic factors related to mandibular biotype, degree of displacement, and type of condylar fracture in association with gender and age. Despite the statistically significant evidence, further studies should be conducted for more robust results, especially with more homogeneous and controlled samples that allow for more concrete conclusions and greater generalization.

## Data Availability

No datasets were generated or analysed during the current study.
